# Supplementary material for: Continuous ultrafiltration during extracorporeal circulation and its effect on lactatemia: A randomized controlled trial
Source: PLoS One. 2020 Nov 23;15(11):e0242411. doi: 10.1371/journal.pone.0242411 (PMC7682870; doi:10.1371/journal.pone.0242411)
Supplement: S1 Protocol — (DOC) [file pone.0242411.s002.doc]

# Continuous Hemofiltration During Cardiopulmonary Bypass and Its Effect on Lactatemia (CPB-LACTATE)

**Study Description**

**Brief Summary**

This study aims to analyze the impact of using polysulfone membranes on continuous ultrafiltration with volume replacement in patients undergoing cardiac surgery. In this type of surgery, techniques such as conventional ultrafiltration (CUF) and modified ultrafiltration (MUF) are known for controlling the patient's fluid balance during the procedure. However, there is no scientific evidence on the benefits of continuous haemofiltration with volume replacement and its effect on lactatemia. Method and design: A single center randomized controlled trial, parallel treatment design with patient-blinded to compare outcomes in terms of the lactate clearance rates (quantity/unit of time) of the assigned therapy groups. Participants will be randomly assigned to receive the type of surgery, in order to ensure an unbiased assessment of treatments, randomisation will be performed in eight blocks of five patients. The study groups will be equivalent in all aspects except the procedures they undergo. Participants will be assigned to the first control group without haemofiltration (CG or 1) or one group with haemofiltration using a Polysulfone filter (PG or 2). Data will be collected by a blinded evaluator.

| **Condition or disease** | **Intervention/Treatment** | **Phase** |
| --- | --- | --- |
| Cardiovascular Diseases | Procedure: Polysulfone Filter Procedure: Procedure/ Surgery: without Polysulfone Filter | Not Applicable |

**Detailed Description**

To determine whether continuous ultrafiltration with volume replacement using a polysulfone membrane during Cardiopulmonary Bypass (CPB) in patients undergoing cardiac surgery decreases intraoperative lactatemia.

**Study Design**

Study Type : Interventional (Clinical Trial).

Actual Enrollment : 76 participants.

Allocation: Randomized.

Intervention Model: Parallel Assignment.

Intervention Model Description: The perfusionist's role is essential during the procedure involving Cardiopulmonary Bypass (CPB). This study purpose to know the impact of using polysulfone membranes on continuous ultrafiltration with volume replacement in patients undergoing cardiac surgery. In this type of surgery, techniques such as conventional ultrafiltration (CUF) and modified ultrafiltration (MUF) are known for controlling the patient's fluid balance and blood lactate levels during the procedure.

Masking: Single (Participant).

Primary Purpose: Treatment.

Official Title: Using Polysulfone Membranes on Continuous Ultrafiltration With Volume Replacement in Patients Undergoing Cardiac.

Actual Study Start Date: September 1, 2017.

Actual Primary Completion Date: January 30, 2018.

Actual Study Completion Date: February 28, 2018.

**Arms and Interventions**

| **Arm** | **Intervention/Treatment** |
| --- | --- |
| Experimental: Polysulfone Filter Group.  The purpose of the research is to determine whether, by controlling the patient's hemodilution level and, therefore, the acute anaemia caused by the Cardiopulmonary Bypass (CPB) priming fluid, continuous conventional ultrafiltration (CUF) can decrease serum lactate levels during normothermic CPB by increasing the haematocrit and, consequently, the supply of oxygen to the tissues, and whether the haemofiltration membrane can remove lactate molecules in situations of hyperlactataemia in CPB. | Procedure: Polysulfone Filter.  The intervention for this group consisted using a Polysulfone filter in order to hemofiltration in the procedure involving Cardiopulmonary Bypass (CPB).The perfusionist's role is essential during the procedure, as they control both Cardiac Output (CO) and gas exchange, depending on their action, will affect blood lactate levels. |
| Active Comparator: Control Group.  The purpose of the research is to determine serum lactate levels during normothermic cardiopulmonary bypass procedure (CPB) without continuous hemofiltration of the patient during the CPB. | Procedure: Procedure/ Surgery: without Polysulfone Filter  In this group, no intervention is performed during the procedure involving Cardiopulmonary Bypass (CPB). |

**Outcome Measures**

**Primary Outcome Measures:**

1. Plasma Lactate Level [Time Frame: Baseline]: The lactate level will be measured before Cardiopulmonary Bypass (CPB) in all patients.

**Secondary Outcome Measures**

1. Maximum Plasma Lactate Level [Time Frame: Every 20 minutes from the start of the Cardiopulmonary Bypass (CPB)]: The maximum level of intraoperative lactate will be measured in all patients, whether the polysulfone membrane has been used or not during Cardiopulmonary Bypass (CPB).

2. Plasma Lactate Level [Time Frame: 1 minute after the Cardiopulmonary Bypass (CPB)]: The level of lactate level will be measured in all patients, whether the polysulfone membrane has been used or not at the end cardiopulmonary bypass (CPB).

3. Plasma Lactate Level in the effluent [Time Frame: 1 minute after the Cardiopulmonary Bypass (CPB)]: To determine lactate levels in the effluent in all ultrafiltered patients.

4. Plasma Lactate Level in intensive care unit (ICU) [Time Frame: 24 hours after the Cardiopulmonary Bypass (CPB)]: Lactate level will be measured 24 hours after surgery in ICU stay.

5. Serum potassium Level [Time Frame: Every 20 minutes from the start of the Cardiopulmonary Bypass (CPB)]: Serum potassium level measured in routine analysis blood.

6. Evaluation criteria of mortality and risk profiles of population [Time Frame: 10 minutes before the Cardiopulmonary Bypass (CPB)]: Mortality predicted and operative risk will be measured by scoring systems European System for Cardiac Operative Risk Evaluation (EuroSCORE I) in cardiac surgery in all patients.

7. Hematocrit [Time Frame: Every 20 minutes from the start of the Cardiopulmonary Bypass (CPB)]: Hematocrit measured in routine analysis blood in all patients.

**Eligibility Criteria**

Ages Eligible for Study: 18 Years and older (Adult, Older Adult)

Sexes Eligible for Study: All

Accepts Healthy Volunteers: No

**Criteria**

Inclusion Criteria:

- Patients who signed informed consent.
- Patients not undergoing emergency surgery.
- Surgical procedures performed under normothermic conditions.
- Patients with a minimum time of 40 minutes before decannulation (after the end of myocardial reperfusion, unclamping of the aorta and the end of CPB).

Exclusion Criteria:

- Emergency medical condition in which it is not possible to collect study data.
- Heart condition requiring the use of hypothermia or hyperthermia during CPB.
- Patients without a minimum time of 40 minutes before decannulation (after the end of myocardial reperfusion, unclamping of the aorta and the end of CPB).
- Patients who cannot manage their fluid balance on their own through diuresis prior to CPB.
- Patients who are unable to manage excess volume during the surgical procedure by means of spontaneous or forced diuresis with diuretics (positive cumulative balance despite intravenous bolus of diuretics after 75% of the anticipated duration of CPB according to the course of the surgery).
