# Supplementary material for: Continuous ultrafiltration during extracorporeal circulation and its effect on lactatemia: A randomized controlled trial
Source: PLoS One. 2020 Nov 23;15(11):e0242411. doi: 10.1371/journal.pone.0242411 (PMC7682870; doi:10.1371/journal.pone.0242411)
Supplement: S2 Protocol — (DOC) [file pone.0242411.s003.doc]

**Continuous hemofiltration during extracorporeal circulation**

**1.- Abstract**

Perfusionists are healthcare professionals with a university degree whose work consists of providing the necessary medical care to maintain and control an adequate blood flow rate in patients who undergo cardiovascular surgery in which is necessary to fully or partially replace cardiac and/or pulmonary function in any medical-surgical procedure involving Cardiopulmonary Bypass (CPB).

Morbidity during heart surgery with CPB is largely due to physiological problems resulting from the blood coming into contact with foreign surfaces such as cannulas, tubing and especially the membrane oxygenator, leading to a condition known as Systemic Inflammatory Response Syndrome (SIRS), as well as hyperlactatemia (HL) caused by hemodilution, tissue hypoxia, low flows during CPB and hyperglycemia, among other factors.

**2.- Purpose**

The purpose of the research is to determine whether, by controlling the patient's hemodilution level and, therefore, the acute anemia caused by the CPB priming fluid, continuous conventional ultrafiltration (CUF) can decrease serum lactate levels during normothermic CPB by increasing the hematocrit and, consequently, the supply of oxygen to the tissues, and whether the hemofiltration membrane can remove lactate molecules in situations of HL in CPB.

This will be done by performing arterial and venous blood gas analyses every 20 minutes during CPB to measure blood lactate levels, pH, oxygen delivery (DO_2_), oxygen consumption (VO_2_), oxygen extraction (ERO_2_), venous oxygen saturation (SvO_2_), arterial and venous carbon dioxide difference (△pCO2) and lactate levels in the effluent from the hemoconcentrator.

The perfusionist's role is essential during the procedure, as they control both Cardiac Output (CO) and gas exchange, as well as a number of variables that, depending on their action, will affect blood lactate levels.

**3.- Background and current state of affairs**

Since Dr John Gibbon [1] performed the first heart surgery with cardiopulmonary bypass (CPB) in 1952 to the present day, important technological advances have been achieved, which have made this an increasingly safe technique to use during surgery.

According to the records of the Spanish Society for Thoracic-Cardiovascular Surgery [2], 33,204 procedures were performed in 2013, of which 45.48% were performed with CPB.

CPB is the procedure that supplies oxygenated blood to the systemic circulation while the lungs and the heart remain stopped so that the cardiovascular surgeon can perform various surgical procedures that it would otherwise be impossible to carry out, such as valve replacements and aortic repair, septal defect repair, coronary artery bypass graft, etc.

Before starting CPB a system of cannulas is required to drain the blood from the heart and return it, once oxygenated, to the most suitable artery for its reinfusion in the systemic circulation.

Cannulation of the ascending aorta is the surgeon's first choice and it is necessary to find the healthiest segment in order to avoid cerebral embolism in patients with atheromatous plaques. Other arteries into which the oxygenated blood can be infused are the femoral artery, the subclavian artery and the axillary artery, which are chosen when perfusion via the ascending aorta is not possible due to lesions (aneurysm, dissection) or surgical reinterventions with CPB.

The patient's venous blood is drained by cannulation of the vena cava and right atrium or selective cannulation of the superior and inferior vena cava or the femoral vein. The choice of drainage system depends on the patient's condition.

The venous blood drains into the circuit via polyvinyl chloride (PVC) tubes. From there, once filtered and defoamed, it passes into a polycarbonate reservoir that uses a roller or centrifugal pump to propel the blood to a thin microporous polypropylene hollow fibre membrane that is composed of two phases: a blood phase and a gas phase, which mimic the capillaries of the lungs. This membrane is folded inside a polycarbonate case in such way that it provides a large contact surface area for gas exchange, the operation of which is based on Fick's law of diffusion, which establishes that the rate of diffusion is proportional to the partial pressure gradient of the gas in the direction of diffusion.

Correct cannulation of the patient before starting CPB, as well as the choice of the cardiopulmonary priming solution, allows us to perform the procedure safely, ensuring proper perfusion of the patient's organs and tissues.

Once CPB begins, the tissues are artificially perfused, for which the perfusion pump must be adjusted to a flow rate that is equal to the cardiac output of a healthy adult [3] (2.5 l/min/m^2^). This rate is adjusted throughout the CPB procedure to adapt to the patient's metabolic needs in order to ensure aerobic metabolism of the different organs and tissues, as the acute hemodilution caused by the priming liquid reduces the blood's oxygen content and blood viscosity, consequently causing hypotension.

Traditionally, during CPB, perfusionist nurses managed a series of gasometric, hematic and electrolyte values that, in theory, ensured safe extracorporeal perfusion, correcting them according to the flow of the CPB pump and temperature in order to maintain normal blood pH between 7.35 and 7.45, venous oxygen saturation (SvO^2^) >65 mmHg to ensure tissue perfusion, a hematocrit above 22% so as not to decrease the availability of oxygen in the blood, and blood glucose, calcium, sodium and potassium values within the normality.

HL can occur during or after CPB without clinical signs of hypoxia [4], increasing postoperative complications such as renal insufficiency (RI), neurological and respiratory changes and hemodynamic instability and causing acidosis. Consequently, patients require more time on mechanical ventilation, are hemodynamically unstable and more vulnerable to infections, all of which lengthens the patient's stay in the intensive care unit (ICU) and can lead to death [5].

Lactic acid is produced from pyruvic acid in a reaction catalyzed by lactate dehydrogenase and is rapidly buffered by the extracellular bicarbonate to produce lactate, normal serum values of which are lower than 2 mmol/L.

HL can begin according to organ involvement and is classified by whether it is related (type A) or unrelated (type B) to tissue hypoxia [6].

Type A HL is associated with CPB due to acute hemodilution and a deficient oxygen supply [7, 8] and is linked to patient morbidity and mortality in the postoperative period when lactate levels are above 4 mmol/L during the surgical procedure [9], as well as prolonging patients' stay in the Intensive Care Unit (ICU).

Intraoperative HL in heart surgery is observed in between 10% and 21% of patients once CPB begins, persisting until the patient reaches the ICU, and it is related to a higher rate of postoperative complications and mortality.

By correcting the risk factors that cause HL during CPB it is possible to control this metabolic disorder and improve patients' prognostic.

In the Perfusion Unit of the Cardiovascular Surgery Unit of Hospital Universitario Puerta del Mar in Cádiz, surgery with CPB that does not require circulatory arrest is performed under normothermic conditions using an alpha-stat acid-base management strategy, as there is no need to correct the pH values based on the temperature. Similarly, the central venous saturation values (SvO_2_) as an indication of the ratio between oxygen consumption and oxygen supply in relation to the patient's body surface area (DO_2_i), oxygen extraction (ERO_2_) and difference between carbon dioxide tension in arterial and venous blood (△pCO_2_) will be recorded every 20 minutes.

The △pCO_2_ is a very important value that helps the perfusionist detect capillary hypoperfusion in surgical patients during CPB, as a difference in arterial and venous pCO_2_ above 8 mmHg, even with optimum SvO_2_ values of >70%, is related to an increase in intraoperative lactate and a decrease in splanchnic function [10].

But there are intraoperative situations in which HL cannot be controlled during CPB, as is the case of patients with hepatic failure, renal failure, intraoperative blood transfusions and pre-surgical HL. In these patients, the evolution of HL is made worse by longer duration of CPB and hypotension during the procedure [11].

Hemofiltration is performed at two clearly distinct points during heart surgery. During CPB it is called conventional ultrafiltration (CUF) and it depends directly on the content of the venous reservoir in the CPB circuit. Modified ultrafiltration (MUF) is independent of this circuit and is performed once the CPB procedure is finished. Both use a highly permeable polysulfone hollow fibre filter housed in a polyvinyl chloride (PVC) cylinder. A pressure gradient and concentration are used to extract liquids, electrolytes and non-protein-bound solutes with a molecular weight below 50,000 Daltons.

Traditionally, CUF is performed in patients with acute kidney injury (AKI), fluid overload (CPB pump priming fluid, cardioplegic solution) and intraoperative hyperkalemia.

MUF seeks to achieve more effective hemofiltration by hemoconcentrating the patient and reducing inflammatory mediators [12].

The surgical team at Puerta del Mar University Hospital who are taking part in the research usually use a polysulfone membrane throughout the procedure and perform high-flow continuous ultrafiltration (UF), replacing the filtered volume with Prismasol 2® pharmaceutical solution, which contains the following active substances: calcium chloride, dihydrate; magnesium chloride, hexahydrate; glucose monohydrate, lactic acid solution 90%; sodium chloride; potassium chloride and sodium hydrogen carbonate. This solution is also used for priming the CPB circuit, and it must be taken into account in the research that Prismasol 2® solution contains 3 mEq/L of lactate.

Prismasol 2® solution is used as a solution for hemofiltration in the treatment of renal insufficiency and no contraindications with CPB have been reported, since serum potassium and calcium levels are monitored every 20 minutes. The administration of sodium bicarbonate is also eliminated from priming.

Likewise, CUF during CPB is safe thanks to continuous patient monitoring throughout the procedure.

The flow rates recommended by the manufacturer for the replacement solution in hemofiltration and hemodiafiltration are as follows: Adults and adolescents: 500 - 3000 ml/hour.

The use of hemofiltration in adults during CPB procedures has evolved over the years, with studies showing the advantages of CUF or MUF in CPB procedures, such as increased hematocrit with the consequent reduction in blood transfusions, reduction of inflammatory response, improvement of hemodynamic parameters and better neurological outcomes in patients, as well as a shorter postoperative intubation time in the intensive care unit [13].

There is a disparity in the scientific literature regarding HL during and after CPB, and while Soliman R et al. [14] consider that continuous hemofiltration increases postoperative lactate levels and inotropic drug use, Kiziltepe et al. [15] describe it as a safe procedure in adult patients undergoing cardiac surgery.

Perhaps the results of the Soliman group were conditioned by the use of moderately hypothermic CPB and the use of Ringer's lactate solution at a concentration of 27.8 mEq/L for priming the CPB circuit.

In terms of blood purification strategies in cardiac surgery, the debate focuses currently on finding out which hemofiltration method is most appropriate: CUF, MUF or a combination of the two strategies. In children, a combination of both therapies seems to be the most effective strategy [16].

In the Cardiovascular Surgery Unit of Puerta del Mar University Hospital, CUF is the technique performed during CPB. The oxygenator propels the blood into the aortic cannula through a 3/8 tube and through a smaller cannula with a 1/16 diameter into the hemofilter. The flow needed from the oxygenator to maintain an intravascular perfusion pressure in the patient depends on changes in systemic vascular resistance. Thus, in the venous reservoir prior to the oxygenator, the volume of blood recovered to be oxygenated from the cannulas may be lower in the event of systemic vasodilation. This is because the anterograde flow (l/min) in the CPB circuit has to be increased to maintain a constant perfusion pressure. By managing the resistance in the efferent line of the filter, transmembrane pressure is increased. This makes it possible to achieve sufficient ultrafiltration through the high-permeability membrane in a controlled manner, even when the blood flow from the oxygenator is not very high. The replacement fluid is administered into the venous reservoir prior to the blood oxygenator. Thus, the resulting hemodilution can be controlled by serial intraoperative controls, maintaining hematocrit values of about 25%. The goal is to achieve a net balance of zero, although it may vary depending on extra hemodilution due to perfusions and intravenous fluid therapy by the anesthetist. Finally, the volume in the lines and the reservoir undergoes an independent hemoconcentration process without prolonging the surgical procedure, once the patient is decannulated, obtaining a bag of packed red blood cells with a hematocrit of 60%. This method benefits from the advantages of the MUF over CUF without any of its disadvantages, such as delaying the completion of surgery.

Taking into account our experience, normothermic CPB allows much more effective oxygen extraction from the tissues than hypothermic CPB, especially splanchnic tissue, reducing bacterial translocation due to hypoperfusion, improving perfusion and avoiding anaerobic metabolism in distal vascular beds.

We will also analyze the possible beneficial effects of optimal therapy on clinical progress in the ICU in the form of reduced need for blood transfusion, mechanical ventilation for less than 24 hours, reduced need for extracorporeal renal support, reduced need for vasoactive drugs, shorter stay in the ICU, shorter hospital stay and reduced mortality. This analysis will enable us to collect information that will allow subsequent patient follow-up in terms of morbidity and mortality.

Finally, based on the important role of perfusion nursing care in patients undergoing cardiac surgery with CPB, and in view of the lack of scientific production in this respect, we propose to assess patients who undergo CPB by means of NANDA, NIC and NOC nursing terms before and after surgery. There are other taxonomies, but these are the most widely accepted and will be used as follows:

- NANDA diagnosis (North American Nursing Diagnosis Association).
- NOC (Nursing Outcomes Classification) of the patient’s current situation and the outcome we hope to achieve.
- NIC (Nursing Interventions Classification).
- NOC that we have achieved.

The interrelationship between these three taxonomies [17] describes the actual or potential problem that we have detected in the patient and the aspects of that problem that we are attempting or hope to solve with one or more nursing interventions, in which one or more activities needed to solve the problem will also be used.

Diagnoses:

- NANDA Code 0027: “Fluid Volume Deficit”
  - Definition: Decreased intravascular, interstitial and/or intracellular fluid. This refers to dehydration, water alone without changes in sodium.
    - NOC Code 0601:
      - Definition: “Water balance in the intracellular and extracellular compartments of the body”.
        - NIC 4120 “Fluid management”: Promotion of fluid balance and prevention of complications resulting from abnormal or undesired fluid levels.
        - NIC Code 4130: “Fluid Monitoring”: Collection and analysis of patient data to regulate fluid balance.
        - NIC Code 7690: “Laboratory data interpretation”: Critical analysis of patient laboratory data in order to assist with clinical decision making.
        - NIC Code 4180: “Expansion of intravascular fluid volume in a patient who is volume depleted”
- NANDA Code 00016: “Impaired urinary elimination”
  - Definition: Impaired urinary elimination.
    - NOC Code 0503:
      - Definition: “Collection and discharge of urine”.
        - NIC Code 0590: “Urinary elimination management”: “Maintenance of an optimal urinary elimination pattern”.
        - NIC Code 2300: “Preparing, giving and evaluating the effectiveness of prescription and nonprescription drugs”
        - NIC Code 1876: “Management of a patient with urinary drainage equipment”
        - NIC Code 4120: “Fluid management” Promotion of fluid balance and prevention of complications resulting from abnormal or undesired fluid levels.
- NANDA Code 00030: “Impaired gas exchange”
  - Definition: Excess or deficit in oxygenation and/or carbon dioxide elimination at the alveolar-capillary membrane.
    - NOC Code 3350:
      - Definition: Adequacy of blood flow through pulmonary vasculature to perfuse alveoli/capillary unit.
        - NIC Code 3350: “Respiratory monitoring”: Collection and analysis of patient data to ensure airway patency and adequate gas exchange.
        - NIC Code 7690: “Laboratory data interpretation”: Critical analysis of patient laboratory data in order to assist with clinical decision making.
        - NIC Code 6680: “Vital signs monitoring”.
        - NIC Code 3320: “Oxygen therapy”.
- NANDA Code 00029: “Decreased cardiac output”.
  - Definition: “Inadequate blood pumped by the heart to meet the metabolic demands of the body”
    - NOC Code 0401
      - Definition: Unobstructed, unidirectional blood flow at an appropriate pressure through large vessels of the systemic and pulmonary circuits.
        - NIC Code 4064: Circulatory care: mechanical assist device.
        - NIC Code 4030: Blood products administration.
        - NIC Code 7690: “Laboratory data interpretation”: Critical analysis of patient laboratory data in order to assist with clinical decision making.
        - NIC Code 4130: “Fluid Monitoring”: Collection and analysis of patient data to regulate fluid balance.
        - NIC Code 6680: “Vital signs monitoring”
        - NIC Code 4210: “Invasive hemodynamic monitoring”
        - NIC Code 4150: “Hemodynamic regulation”
        - NIC Code 6650: “Surveillance”

**4.- Hypothesis and Objectives**

**4.1.- Rationale:**

The intraoperative use of hemofiltration in cardiac surgery in adults has evolved from its initial indication for renal, metabolic and volume control to an immunomodulatory intention as more pathophysiological knowledge has been gained about the process.

In adults, the benefits of using ultrafiltration versus not doing so are clear, although its use is not very widespread, especially in the case of modified ultrafiltration, with disparate published results and conclusions regarding the degree of immunomodulation that hemofiltration produces and its impact on clinical outcomes.

This work aims to study the impact of using polysulfone membranes on continuous ultrafiltration with volume replacement in patients undergoing cardiac surgery. In this type of surgery, techniques such as conventional ultrafiltration (CUF) and modified ultrafiltration (MUF) are known for controlling the patient's fluid balance during the procedure. However, there is no scientific evidence on the benefits of continuous hemofiltration with volume replacement and its effect on lactatemia.

**4.2.- Hypothesis:**

- Continuous ultrafiltration with volume replacement using a polysulfone membrane during CPB in patients undergoing cardiac surgery decreases intraoperative lactatemia.
- Patients undergoing cardiac surgery and continuous ultrafiltration with volume replacement using a polysulfone membrane have a lower postoperative morbidity and mortality rate than patients undergoing cardiac surgery without hemofiltration.
- Patients undergoing cardiac surgery and continuous ultrafiltration with volume replacement using a polysulfone membrane have a better health status after surgery than patients undergoing cardiac surgery without hemofiltration. (Assessed by NNN taxonomies: NANDA, NOC, NIC).

**4.3.- General objectives:**

- To determine whether continuous ultrafiltration with volume replacement using a polysulfone membrane during CPB in patients undergoing cardiac surgery decreases intraoperative lactatemia.
- To assess morbidity and mortality with the use of CPB and continuous hemofiltration.
- To evaluate the health status of patients before and after surgery with CPB using the NNN taxonomies (NANDA, NOC, NIC).

**4.4.- Specific objectives:**

- To determine plasma lactate levels in patients who undergo CPB at different stages of the process:
  - 10 minutes after starting CPB.
  - Every twenty minutes until the end of the CPB procedure
  - Twenty minutes after stopping CPB.
- To determine lactate levels in the effluent.
- To describe the behavior of variables that may influence intraoperative lactatemia levels.
- To evaluate the effects of using the polysulfone membrane continuously during the intraoperative and postoperative period.
- To describe the epidemiological profile, risk factors and surgical risk, according to the usual scores, of the patients in the study.
- To analyze the complications and side effects deriving from CPB and continuous hemofiltration with volume replacement.
- To identify patients' health status using the NANDA diagnosis classification.
- To assess patients' status of using the Nursing Outcomes Classification (NOC), before and after surgery with CPB.
- To evaluate the efficacy of the Nursing Interventions Classification (NIC), after CPB surgery using the Nursing Outcomes Classification (NOC).

**5.- Methodology**

**5.1.- Study design**

A randomized, patient- and data analyst-blinded clinical trial to compare outcomes in terms of the lactate clearance rates (quantity/unit of time) of the assigned therapy groups. Two treatment groups will be established: one control group without hemofiltration (CG or 1), one group with hemofiltration using a polysulfone filter (PG or 2).

To ensure an unbiased assessment of treatments, randomization will be performed in eight blocks of five patients. The study groups will be equivalent in all aspects except the procedures they undergo.

**5.2.- Scope of the study**

Patients operated on in the Cardiac Surgery Department of Hospital Universitario Puerta del Mar in Cádiz.

**5.3.- Study subjects**

Adult patients of both sexes who undergo cardiac surgery and need intraoperative support from the CPB pump at Hospital Universitario Puerta del Mar in Cádiz between February 2017 and August of the same year.

**5.4.- Description of methods:**

The proposed methods to be used in this study are based on analyses of the following instruments and variables:

- Clinical and analytical instruments.
- Record sheet of variables from the patient's medical record.
- Record sheet of intraoperative variables.
- Graph from the perfusionist and electronic data collection systems.
- Record sheet of postoperative variables.
  - Graph and records from the ICU.
  - Graph and records from the ward.
  - Lactate testing instruments.

The variables to be studied have been selected according to the existing literature regarding the factors associated with the results of the therapy used.

- Dependent variables:
  - Levels of serum lactate in mmol/L (continuous quantitative variable): at the beginning of CPB, after 10 minutes of CPB, every 20 minutes during the procedure and 20 minutes after the end of CPB.
  - Effluent lactate levels in mmol/L (continuous quantitative variable): at the end of the procedure.
  - Intra- or perioperative mortality, yes/no (dichotomous variable).
  - Complications: intubation, seizures, pleural effusion requiring drainage, yes/no (dichotomous variable).
  - Evaluation of the outcome of the intervention by the perfusionist nurse. During CPB. NOC nursing taxonomy in the preoperative period, at the end of each procedure and in the postoperative period. These outcomes consist of indicators that measure the patient's health on a Likert scale (1-5).
- Adjustment or stratification variables:
  - Predicted mortality scale (EuroSCORE[[1]](#footnote-2)).
  - Serum potassium and lactate values, immediately after surgery and with each routine blood analysis in the ICU.
- Preoperative variables:
  - Demographic variables:
    - Age: years (quantitative variable).
    - Sex: male/female (dichotomous variable).
- Biometric variables:
  - Height: centimeters (continuous variable).
  - Weight: kilograms (continuous variable).
    - The above variables will be used to calculate the body mass index (BMI): weight/height^2^ = kilograms/m^2^ (continuous variable).
- Biochemical variables:
  - Glucose: mg/dL (continuous variable).
  - Serum lactate: mmol/L (continuous variable).
  - Diagnosis upon admission: according to ICD-9 international classification.
    - Comorbidities: presence/absence in the patient's medical record (dichotomous variables):
      - Arterial hypertension.
      - Hyperlipidemia.
      - Peripheral artery disease.
      - Chronic obstructive pulmonary disease.
      - Impaired renal function.
      - Impaired hepatic function.
      - Type 2 diabetes mellitus.
      - Cerebrovascular disease.
      - Pulmonary hypertension.
    - Myocardial function prior to surgery:
      - Left ventricular dysfunction (whether or not due to previous myocardial infarctions), measured according to the ejection fraction (EF) described in the most recent echocardiography/ventriculography and categorized into:
        - Normal EF > 55%.
        - Mildly impaired function 45-55%.
        - Moderately impaired function 30-45%.
        - Severely impaired function <30%.
        - Functional assessment of heart failure (ordinal variable: NYHA (New York Heart Association) functional class I to IV.
    - Existence of previous liver disease: yes/no (dichotomous variable).
    - Drug allergies: yes/no (dichotomous variable).
    - Smoking history: yes/no (dichotomous variable). If “yes”: current number of cigarettes/day (quantitative variable).
    - Postoperative renal failure: yes/no (dichotomous variable).
    - Classification of acute kidney injury according to the AKIN classification: stages 1/2/3 according to serum creatinine and diuresis (categorical variable).
    - Risk stratification: the EuroSCORE logistical I [21] (Annex I) will be used for preoperative surgical risk assessment: categorical variable.
    - Medical treatment and dosage: yes/no (dichotomous variable) and milligrams/day, of:
      - Antihypertensives.
      - Diuretics.
      - Beta-blockers.
      - Calcium channel blockers.
      - Digitalis.
      - Antiplatelet therapy and withdrawal time prior to surgery.
    - Complexity (categorical variable):
      - Non-coronary surgery.
      - Coronary surgery, single procedure.
      - Coronary surgery, two procedures.
      - Coronary surgery, three or more procedures.
      - Surgery on thoracic aorta.
- Intraoperative variables:
  - Anaesthetic variables:
    - Induction and maintenance: active substances (categorical variable).
    - Volume used in anaesthetic induction prior to CPB: ml (continuous variable).
    - Monitoring: Use of pulmonary artery catheter, yes/no (dichotomous variable).
    - Use of vasoactive and inotropic agents: drugs (categorical variable) immediately after coming off the pump and upon arrival in the ICU.
    - CPB or extracorporeal perfusion variables:
      - Blood products:
        - Haemoglobin in mg/dl and baseline haematocrit measured at the start of CPB (continuous quantitative viable). Use of blood products in priming and/or during the procedure, yes/no (dichotomous variable).
      - Additives:
        - Use of corticotherapy, yes/no (dichotomous variable).
        - Use of insulin, yes/no (dichotomous variable).
      - Times:
        - Surgery time, in minutes (continuous variable).
        - Aortic cross-clamping time in minutes (continuous variable).
        - Support time, in minutes (continuous variable).
        - Haemofiltration time, in minutes (continuous variable).
        - Time from unclamping to patient decannulation, in minutes (continuous variable).
      - Haemofiltration:
        - Duration, in minutes (continuous variable).
        - Total volume (VTot) extracted in the effluent and Replacement volume (VolR) with plasma solution during CPB, in thousands (continuous variable).
        - Water balance during CPB (VTot-VolR), in millimeters (continuous variable).
      - Metabolic and homeostatic control:
        - Blood gas, acidosis yes/no (dichotomous variable).
        - Glucose, in mg/dl (continuous variable).
        - Temperature, in degrees centigrade (continuous variable).
        - Anion gap in mEq/L (in order to diagnose potential lactic acidosis in cases of poorly-defined metabolic acidosis with levels above 12 mEq/L) [22] (continuous variable).
  - Surgical variables:
    - Surgical procedure performed.
      - Valve surgery, yes/no (dichotomous variable).
      - Coronary surgery, yes/no (dichotomous variable).
      - Combined surgery, yes/no (dichotomous variable).
  - Postoperative variables in ICU.
    - Clinical and scale variables:
      - Intubation time, in hours. (continuous variable).
      - Time of stay in ICU, in hours. (continuous variable).
      - Number of hours on inotropic and/or vasoactive support. (continuous variable).
      - Blood loss.
        - Transfusion of blood products, yes/no (dichotomous variable).
        - Postoperative hospital stay, in days (quantitative variable).
        - Hospital mortality, death yes/no (dichotomous variable).

Hematocrit in percentage (continuous variable).

SOFA score [23] (Annex II) (categorical variable).

**5.5.- Choice of sample**

**5.5.1.- Inclusion criteria:**

- Patients who sign the informed consent form.
- Patients not undergoing emergency surgery.
- Surgical procedures performed under normothermic conditions.
- Patients with a minimum time of 60 minutes before decannulation (after the end of myocardial reperfusion, unclamping of the aorta and the end of CPB).

**5.5.2.- Exclusion criteria:**

- Patients who do not sign the informed consent form.
- Emergency medical condition in which it is not possible to collect study data.
- Heart condition requiring the use of hypothermia or hyperthermia during CPB.
- Patients without a minimum time of 60 minutes before decannulation (after the end of myocardial reperfusion, unclamping of the aorta and the end of CPB).
- Patients who cannot manage their fluid balance on their own through diuresis prior to CPB.
- Patients who are unable to manage excess volume during the surgical procedure by means of spontaneous or forced diuresis with diuretics (positive cumulative balance despite intravenous bolus of diuretics after 75% of the anticipated duration of CPB according to the course of the surgery).

**5.6.- Sample size**

The sample size will result from the prospective inclusion of a consecutive series of patients over 10 months who undergo surgery with CPB by the investigator in the Cardiovascular Surgery Unit of Puerta del Mar University Hospital, Cádiz, under normothermic conditions. As a preliminary analysis, this cohort is estimated to consist of about 40 patients in the Cardiovascular Surgery Unit.

The study population will comprise a sample of patients who meet the inclusion criteria between February 2017 and December 2017.

**5.7.- Statistical analysis [24]:**

- Descriptive analysis of variables: measures of central tendency and dispersion will be shown in the case of quantitative variables, while frequencies and proportions will be shown in the case of categorical variables.
- The normality of the variables will be checked using graphs and the Kolmogorov-Smirnov test.
- Homogeneity and comparability between the groups of the two arms of the study in the main confounding factors considered will be verified at the beginning of the study by means of a comparison of proportions (chi-squared test or Fisher's exact test, if necessary) and a comparison of means (Student's t-test and the Mann-Whitney U test in case of non-parametric distributions).
- The intrasubject change over time will be analyzed by comparing paired means using a t-test or Wilcoxon signed rank test (non-parametric variables), quantifying the change in each measure in relation to the baseline reference measure (first sample).
- The bivariate analysis between the CG and PG will be performed using the chi-squared test or Fisher's exact test, if necessary, and using a comparison of means (Student's t-test and Mann-Whitney U test in case of non-parametric distributions).
- In the event of non-homogeneity in any variable between the groups studied, a multivariate analysis will be performed using linear regression.

**5.8.- Limitations of the study and ethical considerations**

The study design does not allow the use of total blinding, and the perfusionist is aware of the therapy and characteristics of the membrane at all times. According to the literature and standard practice in this country, the use of a control group should not pose any ethical dilemma if certain premises are taken into account.

The only condition that the control group must meet is that the patients must be able to manage their fluid balance by themselves, as a fluid overload has been shown to be harmful. This may mean that the patients in the control group have better renal function than those in the other group, or at least better initial diuresis. However, the primary objective is not to measure or evaluate perioperative renal function. In addition, a hemofilter will be available for use if control patients are not able to manage their fluid balance through diuresis. Finally, if surgery times are shorter in the control group, the lactatemia levels may be lower than in the other group with a longer duration. If this is the case, we will stratify the treatment groups by selecting cases with a shorter duration. In any case, a minimum time of 60 minutes is established from start of CPB + CUF and to the end of CPB.

It will be carried out according to the international ethical recommendations (the Declaration of Helsinki and the Oviedo Convention), since the use of the best available therapy according to the patients' circumstances is contemplated at all times.

In order to safeguard confidentiality, all personal data collected as part of this study will be treated in accordance with Organic Law 15/1999, of December 13, on personal data protection.

Likewise, informed consent will be requested for surgery that includes the usual CPB procedures with or without hemofiltration.

Informed consent will also be requested for use of any data deriving from medical records and analytical determinations. Access to personal information will be restricted to the investigator and authorized personnel and all of them will be obliged to maintain confidentiality. If the work is published, the patients' identity will remain anonymous at all times.

**6.- Justification of available resources**

In the Cardiovascular Surgery Department of Hospital Universitario Puerta del Mar, where the research will be carried out, we have the necessary equipment to conduct the study, namely:

- Stocker S5® CPB Pump.
- GEM PREMIER 4000® blood gas analyzer.
- Stocker CONNECT® data management system.
- Spectrum Medical M4® online data collection system.
- BIS® Brain Monitoring System.
- Medtronic® HMS coagulation monitor.

In addition to this, we have access to the resources provided by the Biostatistics and Epidemiology of University of Cadiz, which basically consists of computer and statistics software.

**7.- Time frame**

Detailed work plan:

1. Drafting of the research proposal: Completion of the research proposal. Date: March - April 2016.
2. Signing of the institutional commitment document. Date: 26 September 2016.
3. Presentation of the project in the research group / team.
4. Permits requested from the Research Committee of Puerta del Mar University Hospital: 11 November 2016.
5. Request for report from the Research Ethics Committee: 11 November 2016.
6. Preparation of data collection sheet and computer database (Access). December 2016 - February 2017.
7. Start of the study and follow up:
   - Patient screening/recruitment: December 2016 - December 2017.
   - Collection of samples: February 2017 - December 2017.
   - Patient follow-up and data collection from medical records: March 2017 - February 2018.
   - Analysis of the data: December 2018.
   - Writing of results: December 2018.
   - Publication and dissemination of study findings: Since December 2018.

**8.- Ethical and/or biosafety implications of the proposed research**

The application was submitted to the Research Ethics Committee on 11 November 2016.

The proposed research respects the fundamental principles of the Declaration of Helsinki, the European Convention on human rights and biomedicine, the UNESCO's Universal Declaration on the human genome and human rights and with respect to the Oviedo Convention on human rights and biomedicine. For this purpose, the pertinent ethics report will be requested from the Research Ethics Committee.

The confidentiality and anonymity of the patients and professionals of the centers will be taken into account in all phases of the study, in accordance with Basic Law 41/2002, of November 14, regulating patient autonomy and rights and obligations of information and clinical documentation; Organic Law 15/1999, on Personal Protection Data, and the General Health Act, articles 10 and 61, establishing the right to privacy and confidentiality.

Royal Decree 1720/2007 of 21 December, which approves the regulation implementing the Organic Law on Personal Data Protection. This is the regulation that implements Organic Law 15/99 of December 13 on Personal Data Protection, which specifies both the principles of law and the security measures to be applied in information systems. It will be applied throughout the project.

When processing and managing the data during the study phase and the subsequent analysis and dissemination of the results, the investigators will take the necessary measures to protect the data and prevent unauthorized access by third parties.

**9.- References**

**9.1.- Main references**

1. Passaroni AC, Silva MA de M, Yoshida WB. Cardiopulmonary bypass: development of John Gibbon’s heart-lung machine. Rev Bras Cir Cardiovasc órgão Of da Soc Bras Cir Cardiovasc [Internet]. 2015 [cited 2016 Apr 15];30(2):235–45. Available from: http://www.ncbi.nlm.nih.gov/pubmed/26107456

2. Bustamante-Munguira J, Centella T, Hornero F. Cirugía cardiovascular en España en el año 2013. Interventions Registry of the Spanish Society of Thoracic-Cardiovascular Surgery. Cirugía Cardiovasc [Internet]. 2014 Oct [cited 2016 Apr 15];21(4):271–85. Available from: http://linkinghub.elsevier.com/retrieve/pii/S1134009614001727

3. García X, Mateu L, Maynar J, Mercadal J, Ochagavía A, Ferrandiz A. Estimación del gasto cardíaco. Utilidad en la práctica clínica. Monitorización disponible invasiva y no invasiva. Med Intensiva. 2011;35(9):552–61.

4. Svenmarker S, Häggmark S, Östman M. What is a normal lactate level during cardiopulmonary bypass? Scand Cardiovasc J [Internet]. 2006 Jan 12 [cited 2016 Nov 13];40(5):305–11. Available from: http://www.tandfonline.com/doi/full/10.1080/14017430600900261

5. Nichol AD, Egi M, Pettila V, Bellomo R, French C, Hart G, et al. Relative hyperlactatemia and hospital mortality in critically ill patients: a retrospective multi-centre study. Crit Care [Internet]. 2010 [cited 2016 Apr 13];14(1):R25. Available from: http://ccforum.biomedcentral.com/articles/10.1186/cc8888

6. Maia Heredero Valdés D, Vivian Mena Miranda DR, Riverón Corteguera RL. ACIDOSIS LÁCTICA: ALGUNAS CONSIDERACIONES. [cited 2017 May 14]; Available from: http://www.bvs.sld.cu/revistas/ped/vol72_3_00/ped04300.pdf

7. Ranucci M, Carboni G, Cotza M, Bianchi P, Di Dedda U, Aloisio T, et al. Hemodilution on Cardiopulmonary Bypass as a Determinant of Early Postoperative Hyperlactatemia. Lazzeri C, editor. PLoS One [Internet]. 2015 May 18 [cited 2016 Apr 13];10(5):e0126939. Available from: http://dx.plos.org/10.1371/journal.pone.0126939

8. Hajjar LA, Almeida JP, Fukushima JT, Rhodes A, Vincent J-L, Osawa EA, et al. High lactate levels are predictors of major complications after cardiac surgery. J Thorac Cardiovasc Surg. 2013;146:455–60.

9. Demers P, Elkouri S, Martineau R, Couturier A, Cartier R. Outcome with high blood lactate levels during cardiopulmonary bypass in adult cardiac operation. Ann Thorac Surg [Internet]. 2000 Dec [cited 2016 Apr 13];70(6):2082–6. Available from: http://www.ncbi.nlm.nih.gov/pubmed/11156124

10. Habicher M, von Heymann C, Spies CD, Wernecke KD, Sander M. Central Venous-Arterial pCO2 and pH Difference Identifies Microcirculatory Hypoperfusion in Cardiac Surgical Patients With Normal Central Venous Oxygen Saturation: A Retrospective Analysis. J Cardiothorac Vasc Anesth [Internet]. 2015;29(3):646-55. Available from: http://dx.doi.org/10.1053/j.jvca.2014.09.006

11. Inoue S, Kuro M, Furuya H. What factors are associated with hyperlactatemia after cardiac surgery characterized by well-maintained oxygen delivery and a normal postoperative course? A retrospective study. Eur J Anaesthesiol [Internet]. 2001 Sep [cited 2016 Apr 17];18(9):576–84. Available from: http://www.ncbi.nlm.nih.gov/pubmed/11553252

12. Papadopoulos N, Bakhtiary F, Grun V, Weber C, Strasser C, Moritz A. The effect of normovolemic modified ultrafiltration on inflammatory mediators, endotoxins, terminal complement complexes and clinical outcome in high-risk cardiac surgery patients. Perfusion [Internet]. 2013 Jul 1 [cited 2016 Apr 18];28(4):306–14. Available from: http://prf.sagepub.com/cgi/doi/10.1177/0267659113478450

13. Huang H, Yao T, Wang W, Zhu D, Zhang W, Chen H, et al. Continuous Ultrafiltration Attenuates the Pulmonary Injury That Follows Open Heart Surgery With Cardiopulmonary Bypass. Ann Thorac Surg. 2003;76:136–40.

14. Soliman R, Fouad E, Belghith M, Abdelmageed T. Conventional hemofiltration during cardiopulmonary bypass increases the serum lactate level in adult cardiac surgery. Ann Card Anaesth [Internet]. 2016 [cited 2016 Nov 12];19(1):45–51. Available from: http://www.ncbi.nlm.nih.gov/pubmed/26750673

15. Kiziltepe U, Uysalel A, Corapcioglu T, Dalva K, Akan H, Akalin H. Effects of Combined Conventional and Modified Ultrafiltration in Adult Patients. Ann Thorac Surg. 2001;71:684-93.

16. Sever K, Tansel T, Basaran M, Kafali E, Ugurlucan M, Ali Sayin O, et al. The benefits of continuous ultrafiltration in pediatric cardiac surgery. Scand Cardiovasc J [Internet]. 2004 Oct [cited 2016 Apr 20];38(5):307-11. Available from: http://www.ncbi.nlm.nih.gov/pubmed/15513315

17. Park HJ. NANDA-I, NOC, and NIC linkages in nursing care plans for hospitalized patients with congestive heart failure. 2010 [cited 2017 May 14]; Available from: http://ir.uiowa.edu/etd/570

18. Roques F, Nashef SA, Michel P, Gauducheau E, de Vincentiis C, Baudet E, et al. Risk factors and outcome in European cardiac surgery: analysis of the EuroSCORE multinational database of 19030 patients. Eur J Cardiothorac Surg [Internet]. 1999 Jun [cited 2016 Apr 20];15(6):816-22-3. Available from: http://www.ncbi.nlm.nih.gov/pubmed/10431864

19. Takagi H, Umemoto T, All-Literature Investigation of Cardiovascular Evidence (ALICE) Group. Worse long-term survival after off-pump than on-pump coronary artery bypass grafting. J Thorac Cardiovasc Surg [Internet]. 2014 Nov [cited 2016 Apr 20];148(5):1820–9. Available from: http://www.ncbi.nlm.nih.gov/pubmed/24946969

20. Nashef SAM, Roques F, Hammill BG, Peterson ED, Michel P, Grover FL, et al. Validation of European System for Cardiac Operative Risk Evaluation (EuroSCORE) in North American cardiac surgery. Eur J Cardiothorac Surg [Internet]. 2002 Jul [cited 2017 May 14];22(1):101–5. Available from: http://www.ncbi.nlm.nih.gov/pubmed/12103381

21. García-Valentín A, Bernabeu E, Pereda D, Josa M, Cortina JM, Mestres CA, et al. Validación de EuroSCORE II en España. Cirugía Cardiovasc. 2014;21(4):246-51.

22. Ramírez JA. Brecha aniónica plasmática. Arch Argent Pediatr. 2005;103(1):51-6.

23. Jones AE, Trzeciak S, Kline JA. The Sequential Organ Failure Assessment score for predicting outcome in patients with severe sepsis and evidence of hypoperfusion at the time of emergency department presentation. Crit Care Med [Internet]. 2009 May [cited 2016 Apr 20];37(5):1649–54. Available from: http://www.ncbi.nlm.nih.gov/pubmed/19325482

24. Argimon Pallás JM (Josep M, Jiménez Villa J. Métodos de investigación clínica y epidemiológica. Elsevier; 2004.

**9.2.- Comment on the most important literature references:**

1. In this article Passaroni provide a general review of the development of CPB, from the first extracorporeal oxygenation devices to the current techniques, referring to the complications of the procedure such as hemolysis and systemic inflammatory response. It is worth noting the reference made to Keyser's study on the two types of CPB pump, roller (occlusive) and centrifugal (non-occlusive), in which no difference was found between the two types.
2. Bustamante-Munguira presents an anonymous registry of cardiovascular surgical in Spain in 2013. They review the activity of 56 centers in this country. It should be stressed that Andalusia, with the greatest population, is third in terms of number of surgical procedures with CPB.
3. Review used to gain insight into the normal cardiac output of a healthy adult patient.
4. In this article, in a retrospective study of 5,121 patients, Svenmarker reports the occurrence of hyperlactatemia with no apparent clinical signs, indicating tissue hypoperfusion.
5. In this multicenter study, Nichol correlates serum lactate levels above 0.75 mmol/L with an elevated risk of mortality in critically ill patients.
6. The author points out the two types of lactic acidosis and how to identify them.
7. Ranucci et al. describe acute hemodilution due to CPB circuit priming, which results in the nadir hematocrit, with decreased oxygen supply to the organs that is associated with hyperlactatemia and poor outcomes in cardiac surgery.
8. Hajjar et al. conclude in their study that hyperlactatemia maintained for six hours after the patient's admission to the intensive care unit is an independent risk factor for poor outcomes in adult patients following cardiac surgery.
9. Demers correlates serum lactate levels above 4 mmol/L during CPB with increased morbidity and mortality.
10. Habicher describes that if there is a △pCO2 greater than or equal to 8 mmHg with a venous saturation equal to or greater than 70%, it increases postoperative lactate levels and decreases splanchnic function.
11. Inoue suggests that the occurrence of lactic acidosis is related to hypotension at the start of CPB.
12. Papadopoulos concludes in his study that MUF has benefits due to the elimination of endotoxins and terminal complement factor, as well as significantly reducing lactate concentrations.
13. Huang concludes in his study that the use of CUF and MUF reduces the harm caused by CPB.
14. This interesting study by Soliman claims that CUF increases lactate levels and the need for inotropic drugs and advises limits on this practice to patients with impaired renal function, poor volume management, decreased response to diuretics and CPB time longer than 2 hours. It advises against continuous CUF throughout the procedure.
15. Unlike Soliman, Kiziltepe recommends the combination of CUF and MUF, stating that it is effective and safe to use in adult patients undergoing cardiac surgery, improving hemodynamics, hemostasis and lung function, also recommending the use of combined UF in high-risk adult patients.
16. Sever demonstrates in this study that MUF decreases the duration of mechanical ventilation, the length of stay in the intensive care unit and the need for blood transfusion and improves postoperative hemodynamics. It is associated with an increase in hemoglobin, hematocrit and platelet levels, and Sever concludes that MUF attenuates the inflammatory response by decreasing levels of inflammatory mediators.
17. The article presents a summary of a dissertation that focuses on linking the North American Nursing Diagnosis Association (NANDA) taxonomy, Nursing Outcomes Classification (NOC), and Nursing interventions (NIC) in the clinical setting for clinical decision making and clinical research.
18. EUROSCORE I: Risk factors contribute to cardiac surgery mortality in Europe. This information can be used to develop a risk stratification system for predicting hospital mortality and assessing the quality of care.
19. A meta-analysis of 22 studies, which recruited a total of 100,000 patients, showed that coronary surgery without CPB is likely to be associated with worse long-term survival (≥5 years) compared to coronary surgery with CPB.
20. The use of the euroSCORE is recommended as a simple system of risk stratification in Europe and America, despite demographic differences.
21. This study reveals the shortcomings of both the euroSCORE and euroSCORE II scales due to poor calibration of both scales.
22. Clinical application of the anion gap.
23. The SOFA score potentially offers valuable prognostic information on survival when applied to patients with severe sepsis with signs of hypoperfusion at the time of admission to the emergency room.
24. A practical reference guide for studying the fundamentals of the scientific method and, specifically, the design and development of a study protocol and the reading and critical interpretation of a scientific article.

**10. Supplementary references**

The following list of references (not essential to the work) includes aspects on hemofiltration and lactatemia during CPB.

1. Maillet J-M, Le Besnerais P, Cantoni M, Nataf P. Frequency, risk factors, and outcome of hyperlactatemia after cardiac surgery. Chest. 2003;123(5).

2. Kogan A, Preisman S, Bar A, Sternik L, Lavee J, Ateret @bullet, et al. The impact of hyperlactatemia on postoperative outcome after adult cardiac surgery.

3. Park TK, Yang JH, Jeon K, Choi S-H, Choi J-H, Gwon H-C, et al. Extracorporeal membrane oxygenation for refractory septic shock in adults. Eur J Cardiothorac Surg [Internet]. 2015 Feb [cited 2016 Apr 14];47(2):e68–74. Available from: http://www.ncbi.nlm.nih.gov/pubmed/25425551

4. Litmathe J, Boeken U, Bohlen G, Gursoy D, Sucker C, Feindt P. Systemic inflammatory response syndrome after extracorporeal circulation: a predictive algorithm for the patient at risk. Hell J Cardiol HJC = Hellēnikē Kardiol Ep [Internet]. Jan [cited 2016 Apr 14];52(6):493–500. Available from: http://www.ncbi.nlm.nih.gov/pubmed/22143012

5. Mandak J, Pojar M, Cibicek N, Lonsky V, Palicka V, Kakrdova D, et al. Impact of cardiopulmonary bypass on peripheral tissue metabolism and microvascular blood flow. Perfusion [Internet]. 2008 Nov [cited 2016 Apr 14];23(6):339-46. Available from: http://www.ncbi.nlm.nih.gov/pubmed/19454562

6. Bendjelid K, Treggiari MM, Romand J-A. Transpulmonary lactate gradient after hypothermic cardiopulmonary bypass. Intensive Care Med [Internet]. 2004 May [cited 2016 Apr 14];30(5):817-21. Available from: http://www.ncbi.nlm.nih.gov/pubmed/14985958

7. Boldt J, Piper S, Murray P, Lehmann A. Severe lactic acidosis after cardiac surgery: Sign of perfusion deficits? In: Journal of Cardiothoracic and Vascular Anesthesia [Internet]. Elsevier; 1999 [cited 2016 Apr 15]. p. 220–4. Available from: http://linkinghub.elsevier.com/retrieve/pii/S1053077099900939

8. Gasparovic H, Plestina S, Sutlic Z, Husedzinovic I, Coric V, Ivancan V, et al. Pulmonary lactate release following cardiopulmonary bypass. Eur J Cardio-Thoracic Surg [Internet]. 2007 Dec [cited 2016 Apr 17];32(6):882-7. Available from: http://ejcts.oxfordjournals.org/cgi/doi/10.1016/j.ejcts.2007.09.001

9. Fraser JF. The interpretation of perioperative lactate abnormalities in patients undergoing cardiac surgery. Anaesth Intensive Care. 2012;40(4).

10. Braun J-P, Jakob SM, Volk T, Doepfmer UR, Moshirzadeh M, Stegmann S, et al. Arterio-venous gradients of free energy change for assessment of systemic and splanchnic perfusion in cardiac surgery patients.

11. Parolari A, Alamanni F, Juliano G, Polvani G, Roberto M, Veglia F, et al. Oxygen Metabolism During and After Cardiac Surgery: Role of CPB.

**Annex I**

**EuroSCORE**

EuroSCORE is the European System for Cardiac Operative Risk Evaluation. It is the most reliable system for obtaining a preliminary estimation of the risk of cardiac surgery.

There are two modes of EuroSCORE:

- The additive EuroSCORE can be calculated at the patient's bedside, adding points manually.

- The logistic EuroSCORE is more accurate than the additive score and is preferred when the surgical risk is high, but can only be calculated with a computer that has the application installed.

To calculate the additive EuroSCORE: it is sufficient to add up all the following points to obtain the estimated surgical mortality rate, that is to say, the number of patients who die during surgery for every 100 patients who are operated on.

| **Variables** | **Score** |
| --- | --- |
| *Patient-related variables* |  |
| Age in years (for every 5 years or fraction over 60 years) | **1** |
| Female gender | **1** |
| Chronic Obstructive Pulmonary Disease with bronchodilators or corticosteroids | **1** |
| Extracardiac arteriopathy (Claudication of extremities. Carotid occlusion or > 50% stenosis. Previous or planned surgery on the abdominal aorta, carotids or limb arteries.) | **2** |
| Neurological dysfunction: affecting ambulation or day-to-day functioning | **2** |
| Previous cardiac surgery, which required opening of the pericardium | **3** |
| Preoperative serum creatinine > 2 mg/dl | **2** |
| Active endocarditis with antibiotic treatment during surgery | **3** |
| Critical preoperative state, any of the following:  Ventricular fibrillation or tachycardia or aborted sudden cardiac death. Perioperative cardiac massage. Preoperative ventilation before anaesthetic room. Perioperative inotropes. Perioperative intra-aortic balloon pump. Preoperative renal failure (anuria or oliguria <10 ml/hour). | **3** |
| *Cardiac-related variables* |  |
| Rest angina requiring IV nitrates until arrival in the anaesthetic room | **2** |
| Moderate left ventricular dysfunction (EF = 30-50%) | **1** |
| Severe left ventricular dysfunction (EF < 30%) | **3** |
| Recent acute myocardial infarction (< 90 days). | **2** |
| Pulmonary hypertension (systolic pulmonary artery pressure > 60 mm Hg) | **2** |
| *Operation-related variables* |  |
| Emergency: surgery is performed the same day | **2** |
| Cardiac surgery other than or in addition to isolated coronary artery bypass graft | **2** |
| Surgery on the thoracic aorta (ascending, aortic arch or descending) | **3** |
| Post-infarct septal rupture | **4** |

**Annex II**

**SOFA Scale**

The SOFA (Sequential Organ Failure Assessment) system was created in a consensus meeting of the European Society of Intensive Care Medicine in 1994 and revised again in 1996. The SOFA system consists of the daily measurement of multiple organ failure based on the scores for six organ systems. Each organ is classified from 0 (normal) to 4 (the most abnormal), providing a daily score of 0 to 24 points. The goal in developing the SOFA was to create a simple, reliable, continuous score that could easily be obtained at each institution.

SOFA score during the first days of admission to the ICU is a good indicator of prognosis. Both the mean and highest score are particularly useful outcome predictors. Regardless of the initial score, an increase in SOFA score during the first 48 hours in the ICU predicts a mortality rate of at least 50%.

| **SOFA score** | **0** | **1** | **2** | **3** | **4** |
| --- | --- | --- | --- | --- | --- |
| **Respiration^a^**  PaO_2_/FiO_2_ (mmHg)  SaO_2_/FiO_2_ | >400 | <400  221-201 | <300  142-220 | <200  67-141 | <100  <67 |
| **Coagulation**  Platelets 10^3^/mm^3^ | >150 | <150 | <100 | <50 | <20 |
| **Liver**  Bilirubin (mg/dl) | <1.2 | 1.2-1.9 | 2.0-5.9 | 6.0-11.9 | >12.0 |
| **Cardiovascular^b^**  Hypotension | No hypotension | MAP<70 | Dopamine ≤5 or dobutamine (any dose) | Dopamine > 5 or norepinephrine ≤ 0.1 | Dopamine > 15 or norepinephrine > 0.1 |
| **CNS**  Glasgow Coma Scale | 15 | 13-14 | 10-12 | 6-9 | <6 |
| **Kidneys**  Creatinine (mg/dl) or urine output (ml/d) | <1.2 | 1.2-1.9 | 2.0-3.4 | 3.5-4.9 or <500 | >5.0 or <200 |

MAP, mean arterial pressure; CNS, central nervous system; SaO_2_, peripheral arterial oxygen saturation.

^a^PaO_2_/FiO_2_ ratio should preferably be used. If it is not available, the SaO_2_ is used

^b^Vasoactive drugs administered for at least 1 hour (dopamine and norepinephrine μg/kg/min)

**Annex III**

**Nursing Interventions Classification (NIC)**

| NIC NURSING TAXONOMY | | | |
| --- | --- | --- | --- |
|  |  |  | |
| FIELDS:  PHYSIOLOGICAL: COMPLEX | INTERVENTIONS | |  |
|  |  |  | |
| CLASSES |  |  | |
|  |  |  | |
| G: Electrolytes and acid-base management | 1910: Acid-base management 1911: Acid-base management: metabolic acidosis 1913: Acid-base management: respiratory acidosis 1912: Acid-base management: metabolic alkalosis 1914: Acid-base management: respiratory alkalosis 2000: Electrolyte management | |  |
| M: Thermoregulation | 3900: Temperature regulation 3902: Temperature regulation: intraoperative 3800: Hypothermia treatment | |  |
|  |  |  | |
| N: Tissue perfusion management | 4120 Fluid management 4130 Fluids monitoring 4150: Hemodynamic regulation 4140: Fluid resuscitation | |  |

**Annex IV**

**Glossary of terms and abbreviations:**

**BIS:** Bispectral Index for monitoring consciousness in anesthesia and critical care.

**CPB: cardiopulmonary bypass.** Set of cannulas and roller pumps that conduct and propel the patient's blood through an artificial oxygenator to return it oxygenated to the body.

**Decannulation:** withdrawal of the cannulas that extract and return the blood to the body during CPB.

**△pCO_2_**: difference between carbon dioxide tension in arterial and venous blood

**DO_2_**: Oxygen delivery

**ERO_2_**: Oxygen extraction

**SOFA Scale:** Sequential Organic Failure Assessment score.

**EuroSCORE:** European System for Cardiac Operative Risk Evaluation.

**High-flow hemofiltration or ultrafiltration**: Process of filtering blood by convection (using hydrostatic pressure) through a semipermeable membrane to obtain at least 35 ml/kg/h of liquid, which is partially or fully replaced according to the intravascular volumetric needs of the patient as a result of said extraction.

**HL**: Hyperlactatemia

**Immunomodulation:** Maintenance of homeostasis of the immune system and/or inflammatory cascade, avoiding excessive activation or an insufficient response to a stimulus of any nature or even in the absence of one.

**Inflammatory mediators:** The set of biomolecules involved in the spontaneous or induced physiological or pathological inflammatory reaction. They usually have a small and medium molecular size and electrical properties in the presence of the plasma pH according to its PI (Isoelectric Point).

**Polysulfone membrane:** A synthetic polymer used, among other applications, to develop semipermeable membranes that are used in high-flow dialysis and hemofiltration.

**NANDA**: The American nursing association (*North American Nursing Diagnosis Association*) that has been working since 1973 to develop, update and disseminate a classification of nursing diagnoses that is currently a reference worldwide.

**NIC**: Interventions (nursing activities or actions) aimed at achieving an intended purpose, and so in the Nursing Care Process we must define the Interventions needed to achieve the previously established Outcomes Criteria such that the generic intervention will involve several actions.

**NOC**: This stands for the Nursing Outcomes Classification, which was developed at the University of Iowa. This classification is the result of research carried out at the University of Iowa College of Nursing since 1991, which is still ongoing. It orders and classifies outcomes sensitive to nursing intervention, making it possible to assess the quality of care provided and measure the outcomes achieved in patients who are affected by nursing care.

**Myocardial reperfusion**: A process by which blood is directed back into the myocardial muscle before return of spontaneous circulation.

**SIRS:** Systemic inflammatory response syndrome.

**SvO_2_**: Venous oxygen saturation

**VO_2_**: Oxygen consumption

1. EuroSCORE Performing cardiovascular surgery involves a surgical risk for patients, which needs to be assessed. Different mortality prediction systems have been used internationally, but the most widely used in Europe is the model developed in 1995 (EuroSCORE) [18], which was updated in 2011 (EuroSCORE II) [19]. In this country, a validation of EuroSCORE II [20] has been attempted, but given the controversies regarding the use of the model for care purposes, the original system (EuroSCORE) is generally still used. This scale, the logistic EuroSCORE model, acts as a probabilistic model that is useful for determining the risk of hospital morbidity and mortality. [↑](#footnote-ref-2)
