# Supplementary material for: Continuous ultrafiltration during extracorporeal circulation and its effect on lactatemia: A randomized controlled trial
Source: PLoS One. 2020 Nov 23;15(11):e0242411. doi: 10.1371/journal.pone.0242411 (PMC7682870; doi:10.1371/journal.pone.0242411)
Supplement: Attachment — Submitted filename: Response to Reviewers.doc [file pone.0242411.s005.doc]

**Reviewer #2: The authors have significantly improved the manuscript. However, it needs some minor revisions:**

**- Abstract:**

**o Please declare secondary endpoints (Reduction in the intubation time, etc.)**

We have added the main secondary endpoints in the study (reduction in the intubation time and time spent in ICU). We have reduced the introduction section of the abstract in order not to exceed the word limit.

**- Methods section:**

**o Please declare secondary endpoints (Reduction in the intubation time, etc.)**

We have added the secondary endpoints in the study (possible benefits in patients with renal failure and the reduction in the intubation time, time spent in ICU and C-reactive protein levels).

**- Results section:**

**o I think that there is typo in table 2 (Euroscore: 94.5±72.40 in low risk CG group)**

There is no typo. This result with high variability is due to the low number of low-risk patients. We have highlighted this information in the discussion.

**I advise the authors to make an extra effort to reduce the introduction and improve the discussion**

We have reduced the length of the introduction. If you feel that more changes should be made in the introduction and discussion, we would appreciate it if you could point out the paragraphs that need to be modified.

Thank you for your time and considerations.
